# Domestic pigs as potential reservoirs of human and animal trypanosomiasis in Northern Tanzania

**DOI:** 10.1186/1756-3305-6-322

**Published:** 2013-11-09

**Authors:** Louise C Hamill, Magai T Kaare, Susan C Welburn, Kim Picozzi

**Affiliations:** 1 Division of Pathway Medicine and Centre for Infectious Diseases, School of Biomedical Sciences, College of Medicine and Veterinary Medicine, The University of Edinburgh, Chancellor’s Building, 49 Little France Crescent, Edinburgh EH16 4SB, UK; 2 Tanzania Wildlife Research Institute, Arusha, Tanzania

**Keywords:** Trypanosomiasis, Trypanosomosis, Pigs, Tanzania, *T. b. rhodesiense*, Sleeping sickness, HAT, AAT, PCR

## Abstract

**Background:**

Pig keeping is becoming increasingly common across sub-Saharan Africa. Domestic pigs from the Arusha region of northern Tanzania were screened for trypanosomes using PCR-based methods to examine the role of pigs as a reservoir of human and animal trypanosomiasis.

**Methods:**

A total of 168 blood samples were obtained from domestic pigs opportunistically sampled across four districts in Tanzania (Babati, Mbulu, Arumeru and Dodoma) during December 2004. A suite of PCR-based methods was used to identify the species and sub-species of trypanosomes including: Internally Transcribed Sequence to identify multiple species; species specific PCR to identify *T. brucei* s. l. and *T. godfreyi* and a multiplex PCR reaction to distinguish *T. b. rhodesiense* from *T. brucei* s. l.

**Results:**

Of the 168 domestic pigs screened for animal and human infective trypanosome DNA, 28 (16.7%) were infected with one or more species of trypanosome; these included: six pigs infected with *Trypanosoma vivax* (3.6%); three with *Trypanosoma simiae* (1.8%); two with *Trypanosoma congolense* (Forest) (1%) and four with *Trypanosoma godfreyi* (2.4%). Nineteen pigs were infected with *Trypanosoma brucei* s. l. (10.1%) of which eight were identified as carrying the human infective sub-species *Trypanosoma brucei rhodesiense* (4.8%).

**Conclusion:**

These results show that in Tanzania domestic pigs may act as a significant reservoir for animal trypanosomiasis including the cattle pathogens *T. vivax* and *T. congolense,* the pig pathogen *T. simiae*, and provide a significant reservoir for *T. b. rhodesiense*, the causative agent of acute Rhodesian sleeping sickness.

## Background

**Figure 1:**
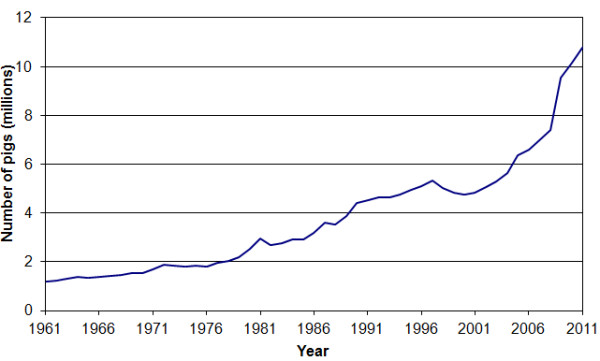
Estimated number of pigs in the East African countries of Burundi, Ethiopia, Kenya, Madagascar, Malawi, Mauritius, Mozambique, Reunion, Rwanda, Seychelles, Somalia, Uganda, Tanzania, Zambia, and Zimbabwe, 1961 – 2011, according to FAOSTAT online database [http://faostat.fao.org].

Around a third of the African continent is affected by trypanosomiasis, of which it is thought around 7 million km^2^ would otherwise be suitable for agricultural development [1]. African animal trypanosomiasis (AAT) is considered to be the principal disease that limits profitable and productive livestock keeping in sub-Saharan Africa [1,2]. Domestic livestock can greatly improve the livelihoods of the rural poor, providing a wealth store that can be drawn on in times of need by selling the animal or animal products [3]. Cattle are traditionally maintained as long term investments for farmers across Sub Saharan Africa, providing draught power, manure, food and transport, which benefits agricultural development and boosts the income of rural households [4]. Cattle trypanosomiasis acts as a major impediment to economic growth in tsetse infested regions, causing severe losses in production due to poor growth, weight loss, low milk yield, reduced capacity for work, infertility and abortion [5]. Over the past decade, domestic pigs have become an increasingly popular livestock strategy in East Africa [6,7] (see Figure 1). Free range pig production is particularly attractive to subsistence farmers as it requires low input and space, pig keeping is considered profitable since there is a perception that they demand few or even zero inputs [8,9]. Both domestic and wild pigs can become infected with various species of tsetse transmitted trypanosomes, but infrequently show symptoms or disease pathology unless infected with *Trypanosoma simiae*, which is highly pathogenic for domestic pigs [10].

Human African trypanosomiasis (HAT) is the collective term for two distinct and fatal parasitic diseases, caused by two morphologically indistinguishable subspecies of *Trypanosoma brucei* s. l.; *Trypanosoma brucei gambiense*, the causative agent of chronic HAT, and *T. b. rhodesiense*, the causative organism of acute HAT [4,11]. HAT causes widespread mortality and morbidity across the tsetse belt of sub-Saharan Africa, with an estimated 1.6 million disability-adjusted life years (DALYs) lost per year [12]. In the 1990s and early 2000s up to 500,000 people were considered infected with HAT with the disease being responsible for some 50,000 deaths per year [13]. In 2009 the number of HAT cases reported fell below 10,000 for the first time in 50 years, largely due to a concerted effort to address the chronic form of the disease (*T. b. gambiense*) led by the World Health Organization (WHO), involving many governments and non-governmental organizations [14].

Significant progress has been made reducing the burden of chronic HAT in rural Africa, which is largely managed through active human screening and case management. However, control of zoonotic *T. b. rhodesiense* HAT remains problematic in the absence of active screening in humans and in the animal reservoir, both of which contribute to under-reporting of the disease in humans and in animals. The long term persistence of both human infective forms in non-human reservoirs further complicates the task of assessing and reducing HAT burden and implementing control measures. These non-human reservoirs of human infective parasites serve to sustain parasite transmission in particular disease foci and maintain HAT even when active screening and treatment of human cases is in place. Persistent infection in cattle outlasts any short-term vector control strategies put in place to deal with acute HAT outbreaks [4].

HAT remains a significant public health problem in sleeping sickness foci in Tanzania. Historical records report both chronic and acute HAT cases around the shores of Lake Victoria [15-17]. Acute *T. b. rhodesiense* HAT is considered to have emerged in the early 1920s in Tanzania [17], although analysis of patient records in the early 1900s suggest that *T. b. rhodesiense* was circulating on the shores of Lake Victoria during the ‘great epidemic’ [15]. Since these early large-scale epidemics the number of reported new cases of HAT in Tanzania has rarely risen above 500 per year and fell to 100 reported cases in 2009 [14,18]. Neither of these figures take into account the extensive levels of under-reporting of sleeping sickness in Tanzania [19] nor do they consider the burden on affected patients, both financially and in terms of DALYs lost [19] and there is concern that *T. b. rhodesiense* HAT will re-emerge as a public health problem as control measures and surveillance are relaxed [18,20]. Public health concerns have been recently heightened by reports of drug resistant *T. b. rhodesiense* isolated from patients in north-western Tanzania [21] and spillover to domestic livestock and humans from wildlife reservoirs of human parasite infection in the National Parks [18]. In the district of Urambo, 98% of reported HAT cases were passively detected, and three quarters of cases were in stage two, suggesting if surveillance was carried out more cases may be detected [19].

Domestic and wild animals have been shown to harbor both *T. b. gambiense* and *T. b. rhodesiense*, but their significance in disease epidemiology remains largely un-quantified. Despite reports of *T. b. gambiense* in domestic (pigs, goats, sheep [22,23]) and wild animals [24], humans are considered the primary reservoir of infection for *T. b. gambiense* HAT [25], responsible for disease persistence since human hosts can maintain an infection for prolonged periods of time [26].

The increasing application of molecular techniques to identify *Trypanozoon* isolates to the sub-species and genotype level now permits critical examination of trypanosome infection in animals and to examine the potential of various host species as reservoirs of the parasites that cause HAT infection. That the same genotype of *T. b. gambiense* has been isolated from humans, pigs and dogs in a HAT focus in the Democratic Republic of Congo implies inter-species transmission in some circumstances [27]. In Cameroon, *T. b. gambiense* was identified in pigs and small ruminants in four active HAT foci in Cameroon [22], the identification of the same *T. b. gambiense* genotype in man and pigs suggesting circulation between human and animals [28,29] and supporting earlier work based on comparison of trypanosome isoenzyme profiles [30]. Tsetse blood-meal analysis also identified *T. b. gambiense* in the midgut of flies that had fed on human and porcine hosts [31].

Mathematical modeling based on data from sleeping sickness foci has implied that *T. b. gambiense* HAT could not be maintained in the focus of Bipindi without the existence of an animal reservoir [32]. However, the outcomes from these models are critically dependent on the input of length of time human and animal reservoir hosts remain infected and that parasites can be picked up by a tsetse fly. Calculating the period between infection and a patient becoming symptomatic is extremely difficult for *T. b. gambiense*. Domestic pigs have been shown to recover from *T. b. gambiense* infection, becoming trypanosome free within a year [33,34].

For *T. b. rhodesiense*, an animal reservoir of infection has long been recognized as epidemiologically significant since humans become infected and die quickly and long-term infection in animal hosts maintains transmission [4]. *T. b. rhodesiense* has been identified in a number of epidemiological surveys that have applied PCR based methods in domestic livestock in East Africa: in cattle in Uganda [35,36], Kenya [37] Tanzania [18]; from numerous wildlife species, in Luangwa valley in Zambia [38,39] and in the Serengeti in Tanzania [39,40]. Domestic cattle are long-term investments and maintain trypanosome infections for prolonged periods of time, acting as long term reservoirs of human infective parasites. Restocking of Zebu cattle in post conflict regions of Uganda has been implicated in the spread of *T. b. rhodesiense* HAT in Uganda, cattle restocking progressively moving the *T. b. rhodesiense* sleeping sickness focus northwards towards that of *T. b. gambiense*[41-43].

While restocking and general cattle movements have contributed to the spread of zoonotic HAT in Uganda, the role of pigs in short term transmission of *T. b. rhodesiense* in smallholder settings in East Africa remains less clear. Pigs are maintained for shorter periods of time but are highly susceptible to infection being frequently exposed to tsetse when free roaming [37]. *Trypanosoma brucei* s. l. was observed in domestic pigs in Uganda in 1984 [44] but identification of human infective *T. b. rhodesiense* was not confirmed until pigs were sampled from three endemic foci in south-east Uganda using the blood incubation infectivity testing (BIIT) [44]. On the southern shores of lake Kyoga, Uganda, *T. b. rhodesiense* infection in pigs is high (1.2 - 14.6%) suggesting that pigs, although short-lived for economic reasons may play a transient role in the epidemiology of HAT [45]. In Busia, Kenya, on the eastern limits of the Ugandan *T. b. rhodesiense* HAT focus, pigs were shown to be at greater risk of *T. b. rhodesiense* infection than either cattle or small ruminants [37].

The aim of this study was to determine the role of pigs as a reservoir of infection for those trypanosomes pathogenic to other livestock (*T. vivax* and *T. congolense* in cattle) and for human pathogenic trypanosomes in the Arusha region of northern Tanzania, using PCR based methods for the detection of parasite DNA.

## Methods

### Samples

**Figure 2:**
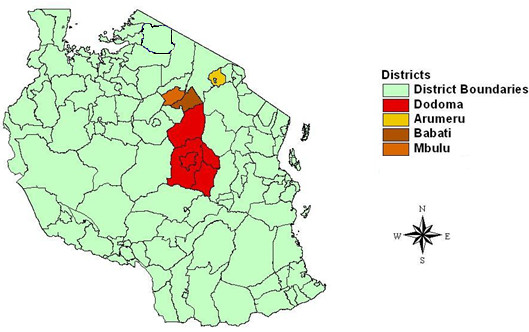
Map of Tanzania, showing districts where pigs were sampled (courtesy of H. K. Auty).

In total 168 domestic pigs were opportunistically sampled across 4 districts in Tanzania during December 2004. These comprised: nineteen pigs from Babati; seventy from Mbulu; thirty-seven from Arumeru and forty-two from Dodoma (see Figure 2). Blood samples were drawn from the jugular vein and were applied directly to Whatman FTA cards following the manufacturer’s guidelines, and air-dried at room temperature for at least 24 hrs prior to being packaged for long-term storage.

### DNA preparation

Five 3 mm discs were taken from each sample and prepared for PCR following the methods previously described [45]. In brief, FTA card discs were washed twice in FTA purification reagent and twice in TE buffer, air dried before being heated at 90°C in the presence of 5% chelex solution [46]. Five microliters of FTA card eluate was used to seed each individual PCR reaction.

### PCR

The following reactions were undertaken to capture the infections present in the pig samples with all reactions using primers and reaction conditions as previously published [47-50]. Internally Transcribed Sequence (ITS-PCR) were used to identify all species that were present in the sample [47]; individual species specific PCR reactions were applied to identify *T. brucei* s. l. [50] and *T. godfreyi*[49], and a multiplex PCR reaction was used to discriminate those human infective *T. b. rhodesiense* from *T. b. brucei*[51] identified from the *T. brucei* s. l. PCR reaction. PCR products were visualized on 1.5% agarose gels run at 100 volts for a minimum of 30 minutes using a 100 bp ladder (supperladder-mid, ABgene, UK) to size the amplified products.

### Statistical analysis

Using graphpadinstat software [52], the 95% confidence interval for each percentage prevalence was computed based on the exact binomial distribution, and displayed in the relevant column in Table 1. Using the same software package the difference between prevalence of the different species and locations were compared using Fisher’s Exact Test; the differences were assigned statistical significance at p values less than 0.05. Fisher’s Exact test was used in the place of the chi squared test due to the small frequencies in some of the categories tested.

**Table 1:** Point prevalence of trypanosomiasis in Pigs (n = 168) by species

**District**	***T. vivax***	***T. simiae***	***T. brucei*** **s. l.**	***T. b. rhodesiense***	***T. godfreyi***
Babati (N = 19)	5.2% [1]*(0.1 – 26.0%)*	0%	0%	0%	5.2% [1]*(0.1 – 26.0%)*
Mbulu (N = 70)	5.7% [4]*(1.6 – 14.0%)*	0%	4.3% [3]*(0.9 – 12.0%)*	1.4% [1]*(0.1 – 7.8%)*	4.3% [3]*(0.9 – 12.0%)*
Arumeru (N = 37)	0%	0%	18.9% [7]*(8.0 – 35.2%)*	10.8% [4]*(3.0 – 25.4%)*	0%
Dodoma (N = 42)	2.4% [1]*(0.1 – 12.6%)*	7.1% [3]*(1.5 – 19.5%)*	16.7% [7]*(7.0 – 31.4%)*	7.1% [3]*(2.7 – 22.6%)*	0%
Overall (N = 168)	3.6% [6]*(1.3 – 7.6%)*	1.8% [3]*(0.4 – 5.1%)*	10.1% [17]*(6.1 – 15.7%)*	4.8% [8]*(2.1 – 9.8%)*	2.3% [4]*(0.7 – 6.0%)*

Where the percentage prevalence is above zero, the actual number of infections detected is given in square brackets, and 95% confidence intervals are given in italics in round brackets.

### Ethical approval

The study was carried out with the full approval of pig keepers and sampling was undertaken with approval of Tanzania National Parks (TANAPA).

## Results

Of the 168 domestic pig blood samples that were analyzed using the four different PCR reactions [47-51], twenty eight (16.7%) were positive for the presence of one or more trypanosome species (Table 1). Seventeen samples (10.1%) tested positive for *T. brucei* s. l*.*; the point prevalence of *T. brucei* s. l. being significantly lower in Mbulu than in Arumeru or Dodoma with P values of 0.0362 and 0.0387 respectively (Fishers Exact Test).

Eight (47%) of the 17 *T. brucei* s. l. positive pigs were identified as harboring human infective *T. b. rhodesiense* giving an overall prevalence of *T. b. rhodesiense* in pigs of 4.8%. Half of the *T. b. rhodesiense* infected animals identified came from the district of Arumeru, where four of 37 animals sampled were infected with *T. b. rhodesiense,* giving a point prevalence of 10.8%.

Application of ITS-PCR detected six cases of *T. vivax* (3.6%), three cases of *T. simiae* (1.8%) and two cases of *T. congolense* Forest type (1.2%) in the 168 pigs sampled; *T. simiae*, pathogenic to pigs, was only found in Dodoma. For *T. vivax* and *T. congolense* (Forest), the principal pathogens of cattle, there were no significant differences between districts.

*Trypanosoma godfreyi* was detected in 4 of the 168 domestic pig samples, generating bands at the expected size of 149 bp; again there were no significant differences between the four districts.

Mixed infections were observed in samples from 2 pigs; one pig being positive for *T. vivax* and *T. congolense* (Forest), and one pig positive for *T. brucei* and *T. simiae*. These pigs were sampled in the districts of Babati and Dodoma respectively.

## Discussion

From this study it is clear that domestic pigs are making a contribution to both AAT and HAT burdens of Tanzanian livestock. Overall the prevalence of trypanosomiasis within the animals screened was 16.7%. Pigs in Arusha are acting as a significant reservoir of infection for those trypanosomes pathogenic to other livestock, *T. vivax* and *T. congolense* in cattle, sheep and goats and for the human infective parasite responsible for acute HAT, *T. b. rhodesiense*.

Overall, in this study ten percent of the pigs sampled were infected with *T. brucei* s. l. while the prevalence of *T. b. rhodesiense* infection in domestic pigs in this survey was high at 4.8%. Pigs sampled in Arumeru, district which lies within a sleeping sickness focus [52], had a very high prevalence of *T. b. rhodesiense* (10.8%).

The unusually high proportion of human infective *T. b. rhodesiense* circulating in domestic pigs, compared with non-human infective *T. b. brucei*, at almost 50% is of concern. Epidemiological theory predicts that where the non-human infective *T. b. brucei* and human infective *T. b. rhodesiense* sub-species coexist, the prevalence in non-humans of *T. b. rhodesiense* should exceed that of *T. b. brucei*[53]. However, numerous field observations using a range of methods to designate human serum resistant *T. b. rhodesiense* suggest the opposite to be true and show that *T. b. brucei* normally predominates in all domestic livestock [18,37,53,54]. The overall ratio of *T. b. rhodesiense* to *T. b. brucei* in domestic livestock species was observed to be 0.35 in all studies of domestic livestock [53], the ratio that was observed in this study in Mbulu district. The very high ratios of human infective *T. b. rhodesiense* to non-infective *T. b. brucei* in pig samples in Dodoma and Arumeru, 0.428 and 0.57 respectively, mirror previous high ratios observed using the blood incubation infectivity test (BIIT) to determine human infectivity, in samples from pigs collected from a sleeping sickness endemic district of Uganda [44]. Elevated ratios of *T. b. rhodesiense* respective to *T. b. brucei* observed in domestic livestock species could be used as a One Health indicator to trigger interventions in livestock to reduce the risk to humans.

A number of studies across East Africa have implicated porcine species in the epidemiology of *T. b. rhodesiense* sleeping sickness; for example, a high prevalence of *T. b. rhodesiense* (9.5%) was identified in warthogs in the neighboring Serengeti district [18] where porcine and bovine species are the preferred hosts for local tsetse populations (*Glossina pallidipes*, *Glossina brevipalpis* and *Glossina swynnertoni*) [55,56]. *T. b. rhodesiense* has been isolated from domestic pigs in western Kenya, on the border of the Ugandan sleeping sickness focus, where *G. pallidipes* are also present [57]. While data from experimental infections in pigs are available for *T. b. gambiense* and *T. b. brucei* from West Africa [33,34], there are gaps regarding *T. brucei* s. l. pathology and pathogenicity in porcine hosts in East Africa including: the length of time that pigs are able to sustain infections with *T. b. brucei* or *T. b. rhodesiense*; the profile of these infections, in terms of parasitaemia, morbidity and mortality in porcine hosts and the longevity of these hosts within the farming system. Pigs are considered to be relatively short lived in small holder farming systems in East Africa [58] but may for short periods of time, contribute a significant pool of infection.

In Tanzania AAT has a large impact on animal health, second only to tick-borne East Coast Fever [59]. Cattle make up the majority of livestock kept in Tanzania and the main pathogenic species to affect cattle are *Trypanosoma vivax* and *Trypanosoma congolense*. While over 50% of the infections identified in pigs in this survey were *Trypanozoon*, and the prevalence of *T. brucei* s. l. was found to be significantly higher than that of any the other species detected, accounting for almost half of the parasites detected (17/38), *T. vivax* (3.6%) and *T. congolense* (1.2%) were also identified in pigs, which is suggestive of low rates of transmission between cattle and domestic pigs. For *T. vivax* and *T. congolense* (Forest) there were no significant differences in distribution between districts, unlike *T. b. brucei* and *T. b. rhodesiense. T. simiae*, that infects pigs, was only found in Dodoma. Sampling of pigs at the village level can be problematic for pig, vet and owner, therefore sampling of pigs at point of slaughter could provide a more straightforward strategy for assessment of HAT risk in Tanzania generally, and enable monitoring of HAT risk at district level.

Attempts in west Africa to control trypanosomiasis in pigs have shown insecticide treated nets to be an affordable and sustainable method for controlling trypanosomiasis in pigsties [60]. Pigs in east Africa are normally free ranging but a move towards simple interventions, such as housing pigs in areas where there is a risk of sleeping sickness could reduce the risk, both for trypanosomiasis, by reducing pig tsetse contact and for other zoonotic diseases (e.g. cysticercosis) by preventing contact of pigs with feces. For example, in Mbulu and Dodoma districts where *T. b. rhodesiense* was identified in pigs, *Taenia solium* cysts have also been observed in a significant number of pig carcasses [58]. Application of insecticide treatments as have been applied to prevent HAT and AAT transmission in cattle in Uganda may also offer opportunities for prevention of transmission of HAT and AAT to and from porcine hosts [61] and could be integrated with methods for control of other endemic zoonotic diseases. Approaches that simultaneously control several zoonotic diseases will amplify the impacts on animal health and pig production and human public health.

## Conclusions

The prevalence of *T. brucei* s. l. in pigs was found to be significantly higher than that of any of the other trypanosome species, accounting for almost half of the parasites detected. That 50% of the circulating *T. b. brucei* s. l. identified in pigs were human infective is of public health concern, in particular the very high prevalence of *T. b. rhodesiense* found in Arumeru district (10.8%), which is within a sleeping sickness focus. The combination of risk factors: tsetse flies that preferentially feed on porcine hosts; pigs with a high prevalence of human infective *T. b. rhodesiense*; a domestic species with a high relative prevalence of *T. b. rhodesiense* to *T. b. brucei* all highlight the importance of monitoring infections in pigs in these districts. Now that PCR based methods can rapidly identify human infective parasites in animal populations, an observed high relative prevalence of *T. b. rhodesiense* to *T. b. brucei* in domestic livestock species could be applied as an indicator to trigger interventions in livestock to reduce risk to the human population.

## Competing interests

The authors declare that they have no competing interests.
